# Management of Hemorrhagic Pseudoaneurysmal Arteriovenous Fistula of the Sphenopalatine Artery

**DOI:** 10.1155/2013/539196

**Published:** 2013-03-27

**Authors:** Ajeet Gordhan

**Affiliations:** Department of Neurointerventional Radiology, Advocate BroMenn Medical Center, 1300 Franklin Avenue, Normal, IL 61761, USA

## Abstract

n-Butyl cyanoacrylate (n-BCA) embolization of a hemorrhagic pseudoaneurysmal arteriovenous fistula of the sphenopalatine artery in a patient with paranasal sinus squamous cell carcinoma treated with regional surgery and radiation has, to our knowledge, not been previously reported.

## 1. Introduction

Pseudoaneurysms of external carotid artery branch vasculature are rare and occur consequent to trauma, infection, iatrogenic injury, and radiation therapy. Concurrent presence of a pseudoaneurysm with an arteriovenous fistula is unusual. This to our knowledge is the first report describing n-butyl cyanoacrylate embolization of a sphenopalatine artery pseudoaneurysmal arteriovenous fistula to arrest active oronasal bleeding in a patient with recurrent paranasal sinus squamous cell carcinoma. 

## 2. Case Report

A 53-year-old female chronic smoker presented to the emergency room with acute onset large volume active oronasal bleeding. She was diagnosed previously with (T4 N2 M0, AJCC Stage IVA, 1997) moderately differentiated invasive squamous cell carcinoma of the left alveolar ridge, left nasal cavity, and maxillary sinus. Gross total surgical resection of the tumor was performed with subsequent radiation and chemotherapy 6 months prior to presentation. Radiotherapy was given by 3 dimensional external-beam radiation with a dose of 68 Gy to the head and neck region. Concurrent chemotherapy consisted of cisplatin. Recurrence of her malignancy was identified 1 month prior to presentation and she was treated initially with Bisphosphonate and subsequently with Carboplatin and Taxol. Her medications included Omeprazole, Acetaminophen/Hydrocodone, Prochlorperazine, and Ibuprofen. She was on self-medicated Ibuprofen of 6400 mg per day for pain relief. Her blood pressure on admission was 134/41 mm of Hg and her heart rate 128 bpm. She was not in apparent distress with normal blood oxygen saturation. On direct inspection, active bleeding within the oral cavity was noted from the the posterior buccal margin of the left maxillary region. Her physical examination was otherwise noncontributory. Her hemoglobin was 10.9 gm/dL, and the hematocrit was 32.4%. Her platelet count was 198 K/uL. A platelet function test was not performed.

**Figure 1:**
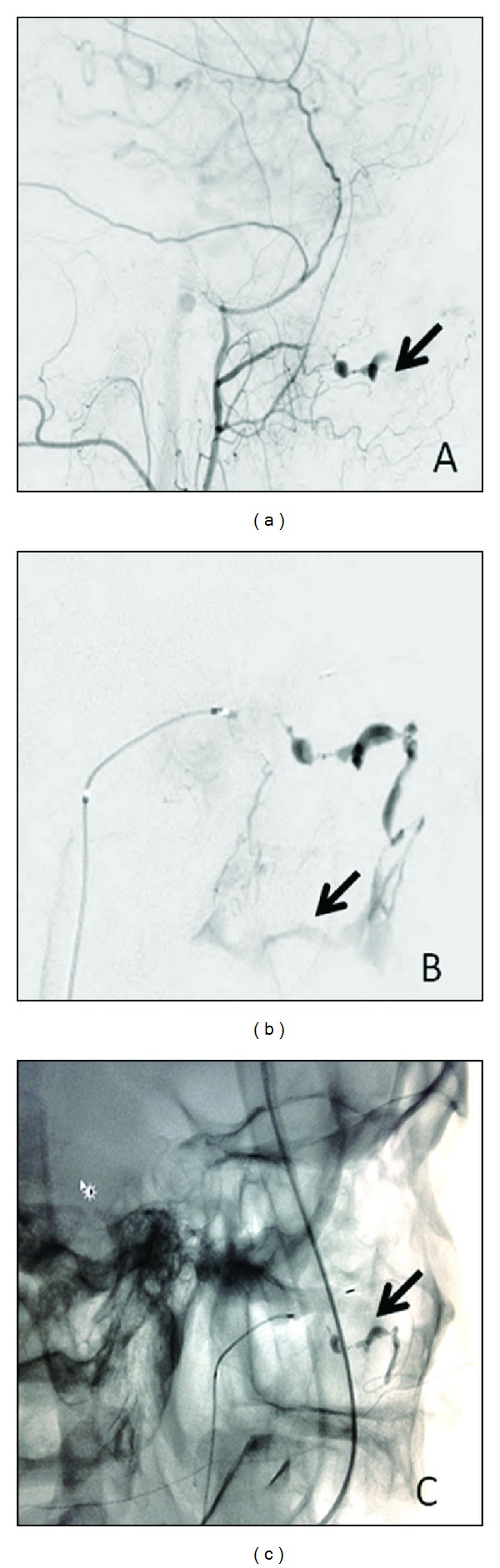
(a) Lateral view digital subtraction angiography of the left external carotid artery demonstrating a pseudoaneurysm of the proximal sphenopalatine artery (arrow). (b) Microcatheter injection digital subtraction angiography of the left internal maxillary artery identifying fistulous venous drainage of the sphenopalatine artery with early venous drainage into the pterygoid plexus (arrow). (c) Unsubtracted fluoroscopic spot view of the maxillofacial region in the lateral view demonstrating the n-BCA cast within the pseudoaneurysm and fistula (arrow).

An emergency room attempt to arrest the bleeding by localized epinephrine injection and direct pressure failed. The bleeding was initially attributed to platelet dysfunction from Ibuprofen usage. Due to ongoing refractory hemorrhage, a catheter-based diagnostic angiogram was requested for. This was performed under endotracheal general anesthesia with access through the right common femoral artery. A 5F MPC guide catheter (Stryker, Kalamazoo, MI) was advanced to the origin of the left external carotid artery using overlaid digital road maps. Digital subtraction angiography revealed a fistulous pseudoaneurysm of the left sphenopalatine artery with early venous drainage into the pterygoid plexus (Figures 1(a) and 1(b)). A Prowler plus microcatheter (Cordis Neurovascular, Inc., FL) was advanced to the proximal aspect of the sphenopalatine artery over a Synchro 14 microguide wire (Stryker, Kalamazoo, MI). Transcatheter n-BCA (Cordis Neurovascular, Inc., FL) admixed in a solution of ethiodal (60 : 40) and tantalum powder was injected (1cc) into the fistula with complete obliteration (Figure 1(c)). Complete cessation of bleeding through the oral cavity was noted immediately after the procedure with no immediate complication.

## 3. Discussion

Aneurysms of the external carotid artery are rare. In a large number of cervical carotid aneurysms, 2.2% were identified to have occurred in the external carotid branch vasculature [1]. External carotid artery aneurysms are predominantly pseudoaneurysms that occur in the context of postsurgical complications, maxillofacial trauma, infection, and irradiation [2–6]. These may present as oronasal hemorrhage, an expanding pulsatile mass with localized compression on neurovascular structures or a source of thromboembolism [7]. Iatrogenic pseudoaneurysms of the sphenopalatine artery have been reported after transsphenoidal surgery for pituitary tumors or maxillofacial surgery and following endoscopic sinus surgery [8, 9]. A pseudoaneurysm can lead to formation of an arteriovenous fistula. Other causes of arteriovenous fistula include arterial dissections, fibromuscular dysplasia, and collagen deficiency syndromes and are rarely congenital [10]. There are very few reports of pseudoaneurysms of the external carotid artery occurring concurrently with fistulous arteriovenous communication [11]. A ruptured fistulous pseudoaneurysm of the sphenopalatine artery induced by surgery and radiation has not been previously described.

Head and neck cancer patients treated with radiotherapy are vulnerable to radiation-induced vasculitis leading most commonly to cervical carotid blowout syndrome [12]. Radiation-vasculitis-induced pseudoaneurysms of the external carotid vasculature are rare [6, 8, 13]. These may present with life-threatening acute uncontrollable hemorrhage [14]. Radiation-induced vascular wall injury is consequent to occlusion of the vasa vasorum and premature atherosclerosis. Permanent fibrosis of the media with focal areas of necrosis in conjunction with periadventitial chronic inflammatory change occurs after the acute phase [15]. A pseudoaneurysm which is essentially a contained hematoma that communicates with the intravascular space occurs after rupture of the endothelium and with blood leakage into and external to the damaged vascular wall. A fragile vulnerable fibrous connective tissue wall forms around the hematoma in 1 to 8 weeks [16]. Irradiation-induced arterial rupture occurs usually within months following treatment and may occur even after a more prolonged period [17]. 

Catheter-based angiography rapidly allows for establishing the etiology and culprit vessel of the blood in addition to facilitating therapeutic intervention. Evaluation by ultrasound may be limited in accessing the distal aspects of external carotid artery branch vasculature [12]. Advanced MR and CT angiographic techniques may allow for the identification of the pathology that incites the blood, that is, fracture or tumor. These noninvasive diagnostic imaging modalities are limited in evaluating the specific angioarchitecture and delay therapeutic intervention in life-threatening hemorrhages [7, 16]. 

Endovascular embolization of internal maxillary artery and related distal branch vessel pseudoaneurysms is favored over surgery because of anatomic and operative complexities [16]. Embolization with various agents to address ruptured and unruptured pseudoaneurysms of the internal maxillary artery has been described [3, 13, 15, 18]. Descriptions of acrylic liquid embolic agent use in the management of pseudoaneurysms without arteriovenous fistula consequent to acute trauma, surgical injury, and radiotherapy of the external carotid artery have been reported [16, 19, 20]. There are no reports describing the use of n-BCA embolization of the sphenopalatine artery for the endovascular management of active oronasal bleeding from a fistulous pseudoaneurysm.

n-BCA is an adhesive liquid embolic agent that allows for rapid delivery into deep complex diminutive vascular anatomy. Its flow characteristics are advantageous in penetrating the focus of the abnormality without direct intralesional catheter placement as would be the requirement with coils. Local delivery of coils may also result in perforation of the fragile false fibrous capsule of a diminutive pseudoaneurysm. A more durable and predictable occlusion can be achieved with the n-BCA than would be with polyvinyl alcohol particle embolization [16]. The rapid vessel occlusion achieved with n-BCA in situations of active hemorrhage is effective even if the patient has platelet dysfunction or a coagulopathy. The risks of liquid embolization of the internal maxillary artery are as with any other embolysate. This includes nontarget delivery resulting in blindness, cranial deficits, and/or cerebral infarction [2]. The most important risk related to n-BCA use is catheter adherence. These can be mitigated by meticulous technique and operator experience. 

## 4. Conclusion

A ruptured pseudoaneurysm with a concurrent arteriovenous fistula of the sphenopalatine artery precipitated by surgery and radiation therapy for squamous cell carcinoma has not been previously described. This is life threatening, and establishment of the diagnosis in the emergent setting with catheter-based angiography is essential. Endovascular embolization with n-BCA is a feasible management option.
